# Relationship between malaria vector survival, infectivity, and insecticide-treated net use in western Kenya

**DOI:** 10.1186/s13071-024-06550-9

**Published:** 2024-11-12

**Authors:** Lucy Abel, Emma Kimachas, Evans Omollo, Erick Nalianya, Tabitha Chepkwony, Joseph Kipkoech, Mark Amunga, Aggrey Wekesa, Jane Namae, Samuel Kahindi, Judith Mangeni, Zena Lapp, Christine F. Markwalter, Steve M. Taylor, Andrew Obala, Wendy Prudhomme O’Meara

**Affiliations:** 1 Academic Model Providing Access to Health Care (AMPATH), Eldoret, Kenya; 2 Duke Global Inc, Nairobi, Kenya; 3 School of Public Health, Moi University College of Health Sciences, Eldoret, Kenya; 4 School of Pure and Applied Sciences, Pwani University, Kilifi County, Kenya; 5 School of Medicine, Moi University College of Health Science, Eldoret, Kenya; 6 Duke Global Health Institute, Duke University NC, Durham, USA

**Keywords:** *Anopheles*, Survival, Blood meal, Infection rates, ITNs

## Abstract

**Background:**

Significant effort and resources have been invested to control malaria transmission in sub-Saharan Africa, but it remains a major public health problem. For the parasite to be transmitted, the female *Anopheles* vector must survive 10–14 days following an infective bite to allow *Plasmodium* gametocytes to develop into infectious sporozoites. The goal of this study was to assess factors associated with wild-caught *Anopheles* survival and infection following host-seeking and indoor resting.

**Methods:**

The study was conducted between January 2020 to March 2022 in a longitudinal cohort of 75 households in 5 villages including a total of 755 household members in Bungoma County, Kenya. Monthly adult mosquito collection was conducted by attenuated aspiration in all enrolled households, and mosquitoes were reared for 7 days. The daily mortality rate was determined through day 7. All mosquitoes were morphologically identified. Female *Anopheles* were dissected, and species-level members of the *Anopheles gambiae* complex were resolved by molecular methods. The abdomens of all samples were processed for *Plasmodium falciparum* oocyst detection by PCR.

**Results:**

Within a 25-month period, the total numbers of non-*Anopheles* and *Anopheles* mosquitoes collected indoors were 12,843 and 712, respectively. *Anopheles gambiae* and *An. funestus* were the major vectors, though their distributions varied between different villages; 61.2% (*n =* 436/712) of the *Anopheles* mosquitoes survived up to day 7, with the lowest mortality rate recorded on day 5 of captivity. The survival rate also varied between the different *Anopheles* species. Six hundred eighty-three of 712 mosquito abdomens were tested for *P. falciparum*; 7.8% (53/683) tested positive for *P. falciparum*, with *An. funestus* having a higher (10%) prevalence than *An. gambiae* s.s. (6.0%, *p =* 0.095, Pearson Chi-square test). The proportion of household members sleeping under a bednet the night before mosquito collection varied across time and village. *Anopheles funestus* survival times were refractory to household ITN usage, and *An. gambaie* s.s. survival was reduced only under very high (100%) ITN usage.

**Conclusions:**

Despite ITN usage, mosquitoes still acquired blood meals and *P. falciparum* infections. Survival differed across species and was inversely correlated with high ITN usage in the household but not oocyst development.

**Graphical Abstract:**

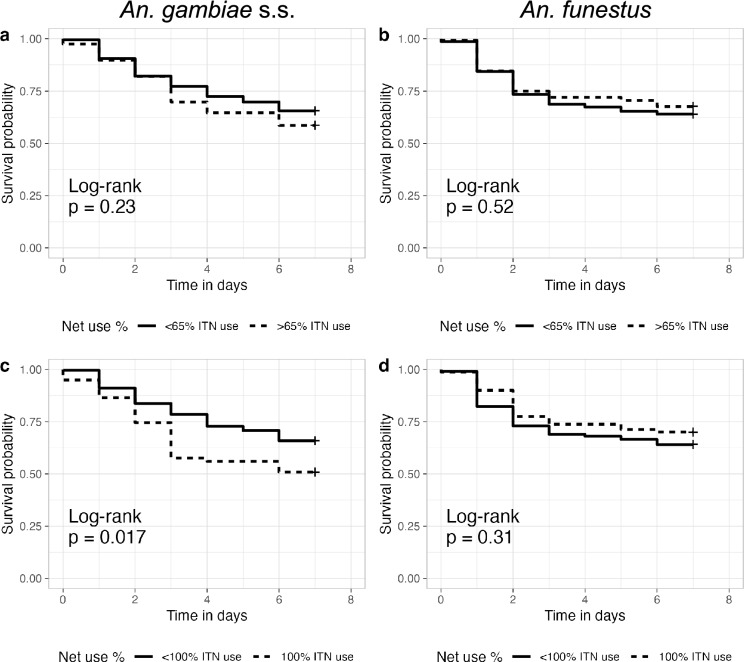

**Supplementary Information:**

The online version contains supplementary material available at 10.1186/s13071-024-06550-9.

## Background

Malaria transmission continues across most of sub-Saharan Africa despite the scale-up of effective vector control methods. *Anopheles* mosquitoes which transmit malaria must survive 10–14 days following an infectious blood meal for the parasite to complete development to the infectious stage (sporozoites) [1]. Therefore, transmission has been predicted to be highly sensitive to the longevity of the vector [2] Vector control tools such as insecticide-treated nets (ITNs) and indoor residual spraying (IRS) are thought to cause mortality among host-seeking or resting mosquitoes and reduce the lifespan of the vector. Such interventions should be effective in reducing transmission if they shorten the average lifespan of the vector population [3] and reduce the number of vectors that survive the extrinsic incubation period.

Malaria transmission is highly dependent on mosquito longevity [4], which is difficult to measure under natural conditions. Standardized assays to estimate vector sensitivity to specific insecticides can only determine chemical resistance and typically observe survival over short time periods after controlled exposures, therefore failing to capture more subtle effects on mosquito survival [5]. However, studying the survival of malaria vectors after natural exposure to ITNs during host-seeking is important for understanding the efficacy of the ITNs.

This study was conducted to measure wild-caught *Anopheles* mosquito survival and infection following host-seeking and indoor resting. The study was conducted within a household-based longitudinal cohort study in Western Kenya [6], where malaria transmission is seasonal and primarily transmitted by *Anopheles gambiae* and *An. funestus*. Resting mosquitoes were aspirated from inside homes and reared for 7 days to investigate the relationship among species, human infectivity to mosquitoes, household ITN use, and mosquito survival. Looking closely at these interdependent factors enables us to understand whether there is a correlation among mosquito infection, mosquito survival and ITN use in western Kenya.

## Methods

### Study area and cohort

The study was carried out in Webuye East and West sub-counties located in western Kenya [6]. The sub-counties are rural, and most families engage in small-scale farming and animal husbandry. Malaria transmission is moderate and perennial, with two seasonal peaks after the long rains (May–June) and the short rains (Sept–October), although the timing and intensity of the transmission peaks can vary from year to year.

From January 2020 to March 2022, we followed a cohort of 755 people aged 1 to 100 years living in 75 households in a rural setting in Webuye, western Kenya. The cohort was assembled in five villages with moderate-to-high malaria transmission using radial sampling of 15 households per village, beginning with a randomly selected household in each village. During the study period, seven households were replaced; three moved and four withdrew.

Face-to-face interviews were conducted by field staff during monthly visits to record information about who slept in the household and bednet use. These included questions such as whether the person had slept under a bednet the previous night, what time they typically go to bed and where they spend time in the evening before going to bed. Those with recent overnight travel were also asked if they had slept under a bednet during their trip.

### Entomological collections

The study team visited each household once per month between 6.00 a.m. and 8.00 a.m. to collect indoor resting mosquitoes via aspiration with Prokopacks (John W Hock Co.). The Prokopacks were fitted with a custom attachment to reduce the aspiration force and decrease damage to mosquitoes during collection. Participants were asked to leave doors and windows closed until the team arrived. Mosquitoes were collected and stored in collection cups inside insulated boxes with 10% sucrose solution dipped in cotton wool attached to each cup with masking tape to feed mosquitoes until they were transported to the insectary laboratory.

In the insectary, we released mosquitoes from each household into individual enclosed cages in a room maintained at 27 °C ± 2 and 80% ± 10% humidity. They were provided with 10% sucrose solution which they could feed on, and this was refreshed every 2 days. Daily mosquito mortalities were recorded from day 0 to day 6. On day 7, all surviving mosquitoes were killed. All mosquitoes were identified morphologically and by PCR.

Mosquitoes were sorted by genus and sex on the day they died. Female *Anopheles* were imaged under magnification for species identification. After photographing the wings, palp and hind leg, female *Anopheles* mosquitoes were dissected, and the head/thorax, abdomen and the wings were stored in separate barcoded tubes packed with desiccant at room temperature. *Anopheles* were identified to the species based on distinguishable characteristics following the Coetzee key [7]. All species identifications were done by two independent observers blinded to the others’ read. Discordant identifications were reviewed by a senior entomologist.

### Molecular analyses

Members of the *An. gambiae* complex were distinguished by PCR [8]. *Anopheles* wing samples were transferred to 96-well plates and extracted using the Hotshot DNA Extraction technique; 50 µl of alkaline lysis solution was added to each well and incubated at 95 °C for 30 min in a PCR machine. An equal volume (50 µl) of neutralizing solution (Tris HCL PH 5.0) was added and stored at − 20 °C. Multiplex species PCR was run on 5 µl of extract using primers specific for *An. gambiae *sensu stricto (463 bp) and *Anopheles arabiensis* (383 bp). Reactions were run on a 1% agarose gel at 100 V for 40 min. DNA fragments were visualized under UV light using SYBR Safe stain added to the agarose gel.

*Anopheles* abdomen samples were processed for *Plasmodium* *falciparum* detection using the technique described in [9, 10]. Briefly, the mosquito parts were ground using a sterile homogenizer in well-labeled microcentrifuge tubes containing 100 µl of 10% saponin. The homogenized samples were transferred to a 96-well plate, and gDNA extraction was done using the simplified Chelex extraction method [11]. A duplex real-time PCR assay targeting pfr364 and human beta-tubulin was performed by amplifying both targets from the samples along with a set of controls at known densities to detect and quantify *P*. *falciparum* in each sample.

Mosquitoes which were identified morphologically as *An. gambiae* s.l. were tested using molecular methods to distinguish *An. gambiae* s.s. and *An. arabiensis*. *Anopheles gambiae* s.l. specimens which did not amplify with primers for *An. gambiae* s.s. or *An. arabiensis* are designated as *An. gambiae* s.l. and are probably another member of that complex.

### Data analysis

Entomological data were recorded on paper forms and then entered into Redcap. Household information was collected and managed using Redcap mobile electronic data capture tools hosted at Duke University [12, 13]. All data were cleaned analyzed using StataSE v17 and visualized using R v4.2.1 (23) in RStudio v2022.12.0 + 353 with the following libraries (tidyverse, dplyr, survminer, ggsurvfit, lubridate).

## Results

Between January 2020 to March 2022, mosquitoes were collected during 25 collection days in 75 households (1875 individual collection events) yielding a total of 12,843 female non-*Anopheles* and 712 female *Anopheles* mosquitoes captured indoors and reared in captivity.

### Distribution of *Anopheles* mosquitoes

**Fig. 1:**
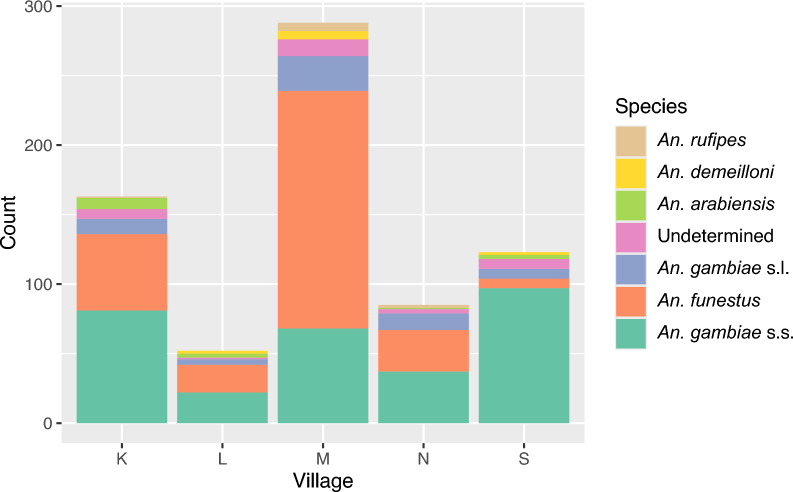
Distribution of *Anopheles* mosquito species by village

*Anopheles gambiae* s.s. (42.9%) and *An. funestus* (39.8%) were the most common vectors, although their relative abundance differed between villages (Table 1, Fig. 1). In one village, most captured vectors were *An. gambiae* s.s. (Village S, 78.9%, Table 1). In contrast, *An. funestus* was dominant in M (59.2%). In the remaining villages, proportions of *An. funestus* and *An. gambiae* s.s. were more even, with *An. gambiae* s.s. slightly outnumbering *An. funestus*. Other mosquito species identified included *Anopheles rufipes 1.3%)*, *An. demeilloni (1.4%)* and *An. arabiensis (2.1%)*, which were captured infrequently.

**Table 1:** Number and proportions of *Anopheles* species captured by indoor resting collection per village

Species	Village					Total
	K	L	M	N	S	
*An. gambiae* s.s	81 (49.7%)	22 (42.3%)	69 (23.9%)	37 (43.5%)	97 (78.9%)	306 (42.9%)
*An. funestus*	55 (33.7%)	20 (38.5%)	171 (59.2%)	30 (35.3%)	7 (5.7%)	283 (39.8%)
*An. gambiae s.l*	11 (6.8%)	4 (7.7%)	25 (8.7%)	12 (14.1%)	7 (5.7%)	59 (8.3%)
*An. arabiensis*	8 (4.9%)	3 (5.8%)	0 (0.0%)	1 (1.2%)	3 (2.4%)	15 (2.1%)
*An. demeilloni*	0	2 (3.9%)	6 (2.1%)	0	2 (1.6%)	10 (1.4%)
*An. rufipes*	1 (0.6%)	0	6 (2.0%)	2 (2.4%)	0	9 (1.3%)
Undetermined	7 (4.3%)	1 (1.9%)	12 (4.1%)	3 (3.5%)	7 (5.7%)	30 (4.2%)
Total	163	52	289	85	123	712

### Female *Anopheles* survival

**Fig. 2:**
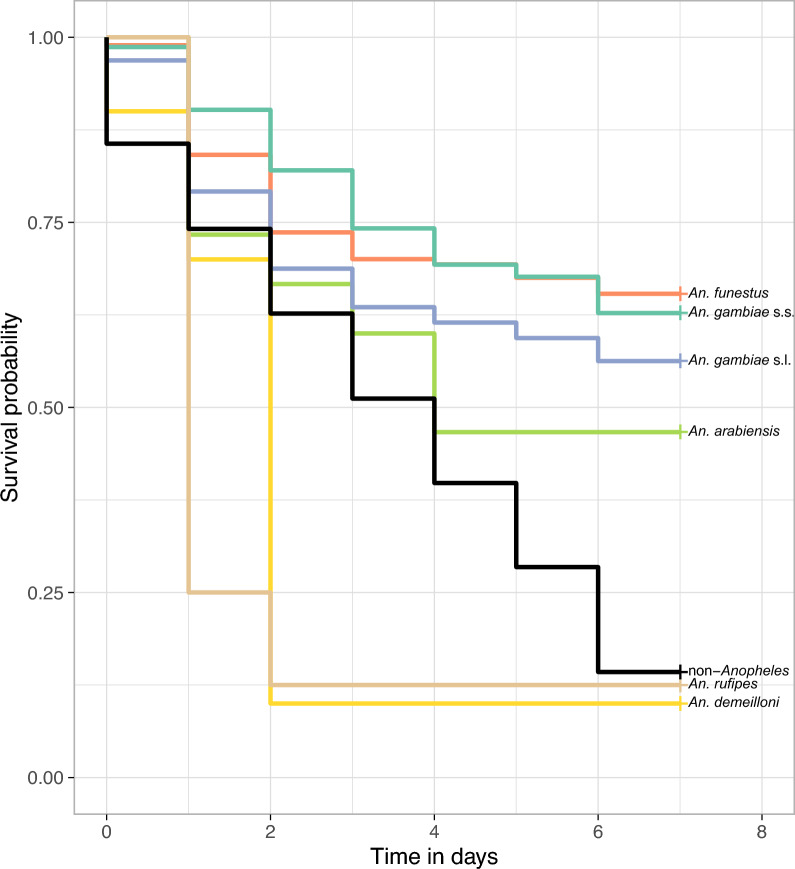
Survival by species from day 0 to day 7

Of the 712 female *Anopheles* captured and reared, 61.2% (*n =* 436) survived up to day 7. The lowest mortality rate was recorded on day 5 of captivity. The number of mosquitoes released into a cage did not impact survival (Additional file 1, Fig. S1). Survivorship to day 7 was slightly different between the vector species (*P =* 0.0005, Fisher’s exact test, Fig. 2), with the lowest mortality rates by day 7 observed for *An. gambiae* s.s. (44%) and *An. funestus* (43%). These two vectors had slightly higher mortality in the first few days compared to days 3–7. The minor vectors had lower survival rates, particularly *An. demeilloni* and *An. rufipes*, which showed very high mortality within the first 2 days of capture. Female non-*Anopheles* mosquitoes showed consistent daily mortality rates with < 15% surviving to day 7.

### *Plasmodium falciparum* infection and *Anopheles* survival

Six hundred eighty-three of 712 *Anopheles* abdomens were tested for the presence of *P. falciparum*. Overall, parasites were detected in 7.8% (53/683) of abdomens. Infection rates differed by species. The *P. falciparum* prevalence was higher in *An. funestus* (10%) than in *An. gambiae* s.s. (6.0%, *p* = 0.095, Pearson Chi-square test; Table 2).

**Table 2:** Abdominal *Plasmodium falciparum* infection by species

Mosquito species	Total tested	Number infected	Oocyst rate per species
*Anopheles gambiae* s.s	298	18	6.0%
*An. funestus*	271	27	10.0%
*An. gambiae* s.l	55	6	10.9%
Undetermined	29	2	6.9%
*An. arabiensis*	15	0	0
*An. demeilloni*	8	0	0
*An. rufipes*	7	0	0
Total	683	53	7.8%

**Fig. 3:**
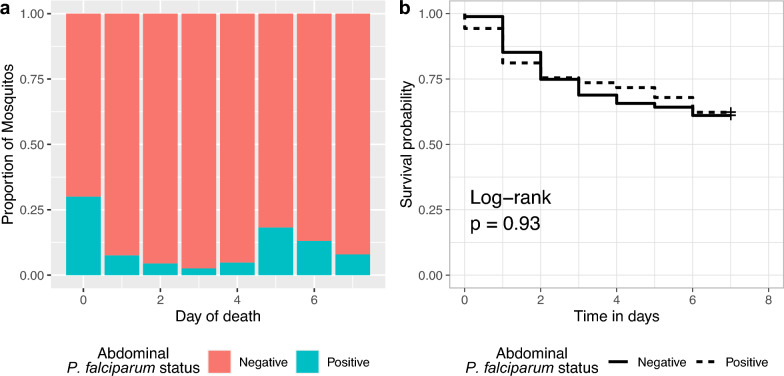
**a** Abdominal *Plasmodium falciparum* infection by day of death and **b** daily surviving proportion by abdominal infection status

The abdominal *P. falciparum* prevalence was highest in vectors that died immediately on day 0 (30%, 3/10; Fig. 3a) followed by those that died on day 5 (18.2%, *n =* 2/11). The *P. falciparum* prevalence was 7.9% (*n =* 33/417) among vectors surviving to day 7. There was no difference in survival among infected and uninfected *Anopheles* (Fig. 3b, log-rank test, *p =* 0.58).

### Bednet use and *Anopheles* survival

**Fig. 4:**
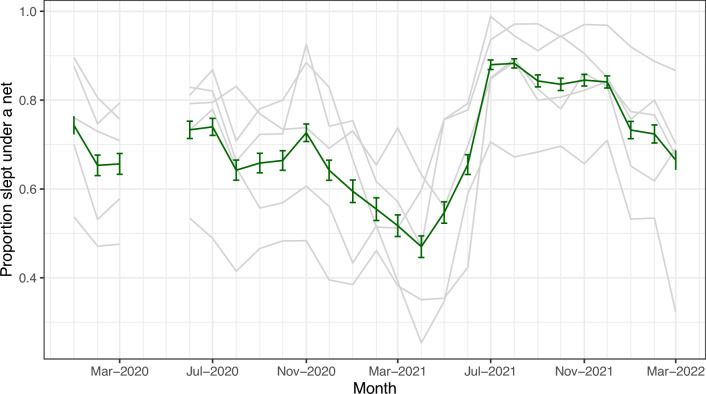
The proportion of people in each of five villages (gray lines) reporting sleeping under a net the night before the survey. The mean and 95% confidence intervals are shown in green. Reported net use rose sharply in June 2021 following a mass net distribution campaign. No data or samples were collected in March and April 2020

The proportion of household members sleeping under a bednet the night before mosquito collection varied across time and village, ranging from 46 to 87% (Fig. 4).

**Fig. 5:**
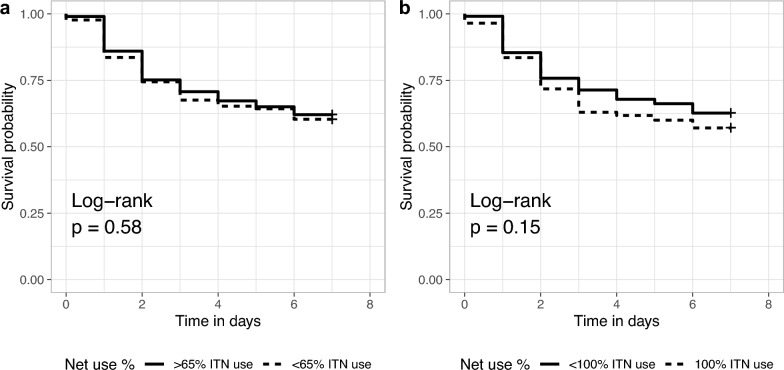
Female *Anopheles* survival between day 0 and day 7 for mosquitoes in households with higher (dotted line) or lower (solid line) ITN usage. **a** Households with at least 65% of members sleeping under an ITN the night before collection compared to less than 65% usage. (**b**) Households with 100% of members sleeping under an ITN compared to less than 100% usage

We calculated the proportion of household members sleeping under an ITN in each household for each day of mosquito collection and correlated ITN usage with the survival of *Anopheles* mosquitoes from that household. There was no difference in *Anopheles* survival when comparing vectors collected from households where at least 65% of the members slept under an ITN versus those with lower net usage. However, when 100% of members slept under an ITN, *Anopheles* collected from those households had lower survival than households with lower usage, although the comparison did not reach statistical significance (Fig. 5, Log rank test *p =* 0.15). In households with 100% ITN usage the night before collection, 56.7% of female *Anopheles* survived to day 7 compared to 62.7% in other households.

**Fig. 6:**
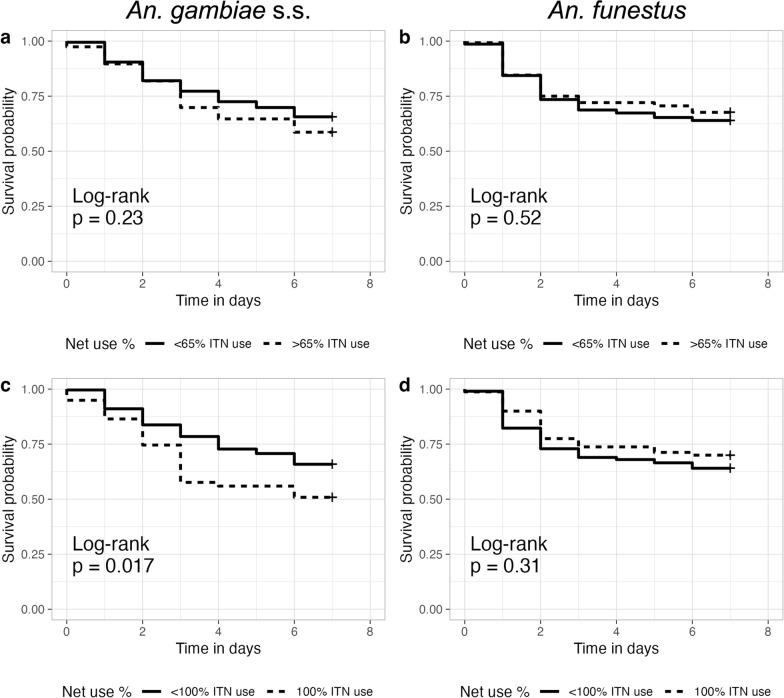
Female *Anopheles* survival between day 0 and day 7 post-collection for mosquitoes collected in households with higher (dotted line) or lower (solid line) ITN usage. **a**, **c** *An. gambiae* s.s. **b**, **d** *An. funestus*

Species-specific survival in households with high or low ITN usage varied. *An. gambiae* s.s. mosquitoes showed greater differences in survival based on ITN usage. *Anopheles funestus* exhibited much less difference in survival to 7 days when captured in households with high ITN usage compared to *An. gambiae* s.s. (Fig. 6).

## Discussion

In this study, we investigated the survival of wild-caught mosquitoes resting in homes in the early morning. These endophilic vectors experienced differing levels of both ITN use and prevalence of malaria-infected hosts [6]. To study the survival of wild vector populations foraging and resting under natural conditions, we reared them in cages for 7 days and recorded daily mortality. We found that most female *Anopheles* survived to the 7th day. Survival differed across species and was inversely correlated with high ITN usage in the household but not oocyst development.

We identified a diverse vector population dominated by two primary vectors, *An. gambiae* s.s. and *An. funestus*. Major vector species had similar survival rates, while minor species exhibited poorer survival following collection. Oocyst infection rates were high for both major vector species and was nearly 8% in those surviving to day 7. This is similar to what was found in western Kenya closer to Lake Victoria and in our previous work in the study area [14]. If we assume an infection prevalence of 30% in the human hosts [15], uniform biting rates, and that 80% of *Anopheles* have fed in the last 24 h [6], then we estimate that one in three bites on an *infected* host must be *infectious* to achieve an 8% oocyst rate.

Regarding the relationship between survivorship and *P. falciparum* oocyst development in malaria vector mosquitoes, we found that infections acquired on or near the day of capture had no effect on survival up to 7 days. Very few studies have assessed how infected or uninfected mosquitoes differ in survivorship, especially for wild-caught mosquitoes. Most studies have been conducted on mosquitoes reared and infected under laboratory conditions. Significant reduction in survival following infection is limited to studies with combinations of vectors and plasmodium species that are not known to occur naturally [16]. The study by Chege and Beier [17] examined the effect of malaria parasites on the longevity of wild-caught, naturally infected *Anopheles*. The results from their study are consistent with our findings that infection status does not affect survivorship. However, in their study they observed higher survival rates and lower infection rates among *An. funestus* compared to *An. gambiae* s.l. A study in Uganda used parity rate as a proxy for longevity and demonstrated that longevity of *Anopheles* mosquitoes collected in villages with ITNs was significantly lower than in villages without [18].

Leveraging detailed ITN use data recorded at every mosquito collection time point per household, we were able to correlate vector survival with ITN usage at the household level. From our study, we found that mosquitoes collected from households that had 100% of its members sleeping under a net the night before mosquito collection had a significantly lower survival rate. However, this effect was not observed at lower ITN usage. In a previous study, we observed that 72% of blood meals taken from household members were on someone not sleeping under a net [19]. Thus, it is likely that mosquitoes taking blood meals in households with lower ITN usage may have capitalized on unprotected blood meal sources and therefore had less exposure to ITNs, resulting in lower mortality. Similar results were reported in an experimental hut system in Burkina Faso where there was no difference in long-term survival of wild-entry *Anopheles* when they entered a hut with an untreated net or a pyrethroid-treated net [20]. Interestingly, blood-fed mosquitoes in the hut studies had significantly better survival than unfed mosquitoes even though the only blood meal source was protected by an ITN. In experimental studies, it has been shown that insecticide-susceptible *Anopheles* can feed across an ITN when a person is touching the net [21]. Although feeding time and blood meals are smaller, only 15% of fed mosquitoes die in the 24 h post-feeding. Given that 80% of the *Anopheles* collected in these study households evidence recent feeding [6], feeding success could partially explain the low mortality after foraging in a household with high ITN usage. The observation that feeding reduces mortality from insecticides has been reported elsewhere [22, 23], but whether this is the cause (the blood meal reduces susceptibility to the chemical) or effect (insects are more successful at feeding if they are less sensitive to the chemical) is difficult to untangle in a natural system such as ours. It is also possible that insects which died quickly after exposure to ITNs were not aspirated from walls, and the collected mosquitoes represent a surviving subset of all foraging mosquitoes. Nonetheless, our findings are consistent with epidemiological studies which show that ITNs reduce incidence of malaria in areas with populations of resistant *Anopheles*, but protection is incomplete and transmission persists [24].

The impact of ITN usage on survival differed by species. While *An. gambiae* s.s. survival was sensitive to ITN usage, *An. funestus* survival did not decline with increasing ITN usage. This could be due to differences in biting behaviors, such as late evening or early morning biting by *An. funestus*, that reduce exposure to ITNs during feeding, or it could be due to higher levels of pyrethroid resistance in *An. funestus* compared to *An. gambiae* s.s., which has been reported elsewhere [25]. Overall, the 48-h mortality rate after foraging and resting in a household with 80 or 90% ITN coverage was < 25%, and differences in survival of *An. gambiae* s.s. exposed to high or low ITN coverage were not noticeable until after day 3. It is unclear whether these small differences in day 7 survival as a function of ITN coverage would reduce overall transmission [3].

Limitations to our study include unknown effects of collection technique on mosquito fitness and survival as well as unknown feeding status, age and life history of the wild-caught mosquitoes. Overall, we observed that vectors had slightly higher mortality in the first few days compared to days 3–7, which might be due to damage during mechanical aspiration of mosquitoes or poor adaptation to cages. Although we reduced the aspiration force of the prokopacks using a bespoke attachment which improves survival, mosquitoes may still suffer damage during collection. In addition, since it is impossible to determine the ages of the harvested mosquitoes, and it is possible that older mosquitoes were more vulnerable to the trauma of aspiration, early mortality may be biased towards older insects. This is supported by the observation that infection rates were higher in mosquitoes that died on the same day they were collected; these mosquitoes must have already survived a minimum of 5–7 days to have been infected on the day of collection. However, since our study was conducted over 24 months, we expect that we collected a representative age distribution of vectors across the study, which increases the generalizability of our findings. Another limitation was that mosquito collection was conducted early in the morning. Therefore, some mosquitoes which contacted the ITNs and died before the collection time may have been missed. In an effort to reduce handling and damage of wild-caught mosquitoes, we were not able to accurately record their abdominal status on the day of collection. We were also not able to analyze the source of the bloodmeal or correlate blood-feeding with survival at the individual insect level. Finally, we cannot rule out the possibility that some mosquitoes bit someone outside the house and moved inside to rest. However, in a previous study from the same households, we observed that 73% of collected blood-fed mosquitoes fed on humans, and 94% of those bit someone in the same household where they were collected [19].

## Conclusions

We report high infection rates among wild-caught vectors. Early stages of parasite infection up to the development of oocysts do not appear to influence survival for the two major vectors identified in this study. In contrast to *An. funestus*, *An. gambiae* s.s. exhibits increased mortality when collected from households with higher ITN usage, although the mortality rate is still lower than would be expected. Rearing wild-caught mosquitoes gives unique insights into factors correlated with vector survival and development of infectivity. Future studies of insecticide resistance and net insecticide levels will provide further insight into the effects of net use on mosquito survival in this natural setting.

## Supplementary Information

Additional file 1. Figure S1. Number of mosquitoes in a collection is plotted against mean survival in days to determine whether the density of mosquitoes in collection cups or cages affected survival. There is no apparent relationship between the number per cage and mean survival of insects in the cage

Additional file 2. Data for Abel Parasites and Vectors 2024. Csv format

## Data Availability

Data can be provided by the corresponding author (W.P.O) upon request.
