# Supplementary material for: Relationship between malaria vector survival, infectivity, and insecticide-treated net use in western Kenya
Source: Parasit Vectors. 2024 Nov 12;17:464. doi: 10.1186/s13071-024-06550-9 (PMC11558830; doi:10.1186/s13071-024-06550-9)

**Relationship between malaria vector survival, infectivity, and insecticide treated net use in western Kenya.**

Supplemental figures

Fig. S1: Number of mosquitoes in a collection is plotted against mean survival in days in order to determine whether the density of mosquitoes in collection cups or cages affected survival. There is no apparent relationship between the number per cage and mean survival of insects in the cage.


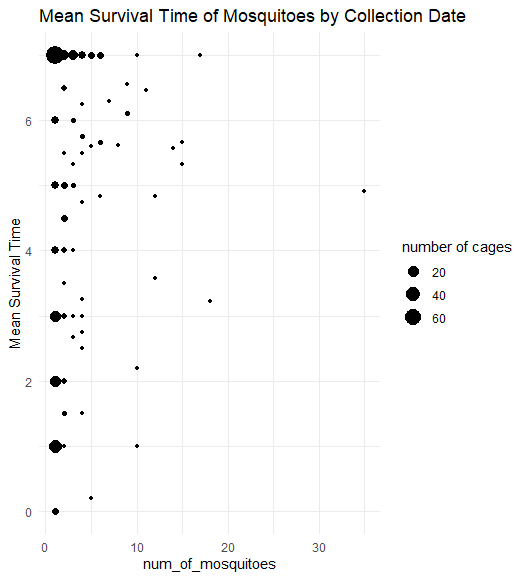

Supplement: Supplementary file 1 — Additional file 1. Figure S1. Number of mosquitoes in a collection is plotted against mean survival in days to determine whether the density of mosquitoes in collection cups or cages affected survival. There is no apparent relationship between the number per cage and mean survival of insects in the cage [file 13071_2024_6550_MOESM1_ESM.docx]
